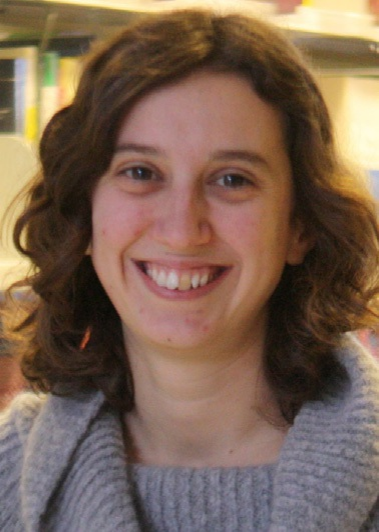# Conformational Studies of Oligosaccharides

**DOI:** 10.1002/chem.202001370

**Published:** 2020-07-09

**Authors:** Yang Yu, Martina Delbianco

**Affiliations:** ^1^ Department of Biomolecular Systems Max-Planck-Institute of Colloids and Interfaces Am Mühlenberg 1 14476 Potsdam Germany; ^2^ Department of Chemistry and Biochemistry Freie Universität Berlin Arnimallee 22 14195 Berlin Germany

**Keywords:** conformations, glycans, structural studies, synthetic oligosaccharides

## Abstract

The conformation of a molecule strongly affects its function, as demonstrated for peptides and nucleic acids. This correlation is much less established for carbohydrates, the most abundant organic materials in nature. Recent advances in synthetic and analytical techniques have enabled the study of carbohydrates at the molecular level. Recurrent structural features were identified as responsible for particular biological activities or material properties. In this Minireview, recent achievements in the structural characterization of carbohydrates, enabled by systematic studies of chemically defined oligosaccharides, are discussed. These findings can guide the development of more potent glycomimetics. Synthetic carbohydrate materials by design can be envisioned.

## Graphical abstract

**Chemical synthesis** and novel analytical techniques have enabled conformational studies of oligosaccharides. Recurrent structural features have been identified. Tuning these structures will allow for the production of more potent glycomimetics and materials by design.

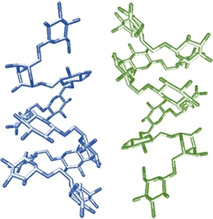

## Introduction

The function of a molecule is strongly connected to its three dimensional shape.^1^ Structural studies of proteins and nucleic acids were fueled by synthetic and gene expression technologies.^2^ Well‐defined synthetic materials serve as standards to establish definitive structure–function correlations.^3^ Analytical techniques, such as X‐ray crystallography^4^ and cryogenic electron microscopy (cryo‐EM),^5^ have enabled subnanometer resolution images. In addition, spectroscopic techniques like circular dichroism (CD) and NMR have made structural studies routine in most chemistry laboratories.^6^ These results permitted the development and validation of highly accurate computational tools that can predict and suggest the fabrication of materials by design (e.g., DNA origami and de novo proteins).^7^

In contrast, structural studies of carbohydrates, the most abundant organic materials in nature, are rare.^8^ Carbohydrates are mainly extracted from natural sources, resulting in ill‐defined mixtures of compounds.^9^ Chemical synthesis offers a valid alternative to isolation, but requires a huge synthetic effort. The advent of one‐pot synthesis and automated techniques has allowed for the access to collections of related compounds, as ideal probes for structural studies.^10^ Oligosaccharides with defined composition, length, and substitution are now available for structural elucidation. Importantly, the insertion of specific modifications, such as NMR active nuclei (i.e., ^13^C and ^19^F) can be easily achieved, offering a tremendous advantage during analysis.10b, 11 Unnatural functionalities able to lock particular conformations or to disrupt particular geometries can be imagined and used for the creation of glycomimetics.^12^ Even though such strategies remain limited by synthetic challenges, examples of structurally designed glycomimetics with high affinity for a target protein suggest the potential of this approach.^13^

An additional bottleneck in the structural analysis of carbohydrates is that standard characterization techniques are often not applicable.8b Carbohydrates’ high flexibility prevent the formation of single crystals suitable for X‐ray analysis, sensitivity to electron beam makes them poor candidate to EM characterization, and lack of chromophores prevents standard CD analysis. NMR remains the most useful characterization technique; however, the analysis is hindered by severe chemical shift degeneracy, often requiring special pulse sequences or the insertion of labels.^14^ Due to the lack of validating standards, computational tools are far less developed as compared to peptides and nucleic acids.^15^ To date, the combination of chemical synthesis, NMR analysis, and molecular dynamics (MD) simulations permitted to identify recurrent structural features common for some glycan classes.13a, 16

Here, we review the recent results obtained in the field of glycan conformational analysis. We discuss four classes of glycans and their structural features. Particular focus is given to the synthetic strategies that were employed to help the characterization.

## Glycans Conformation

**Figure 1:**
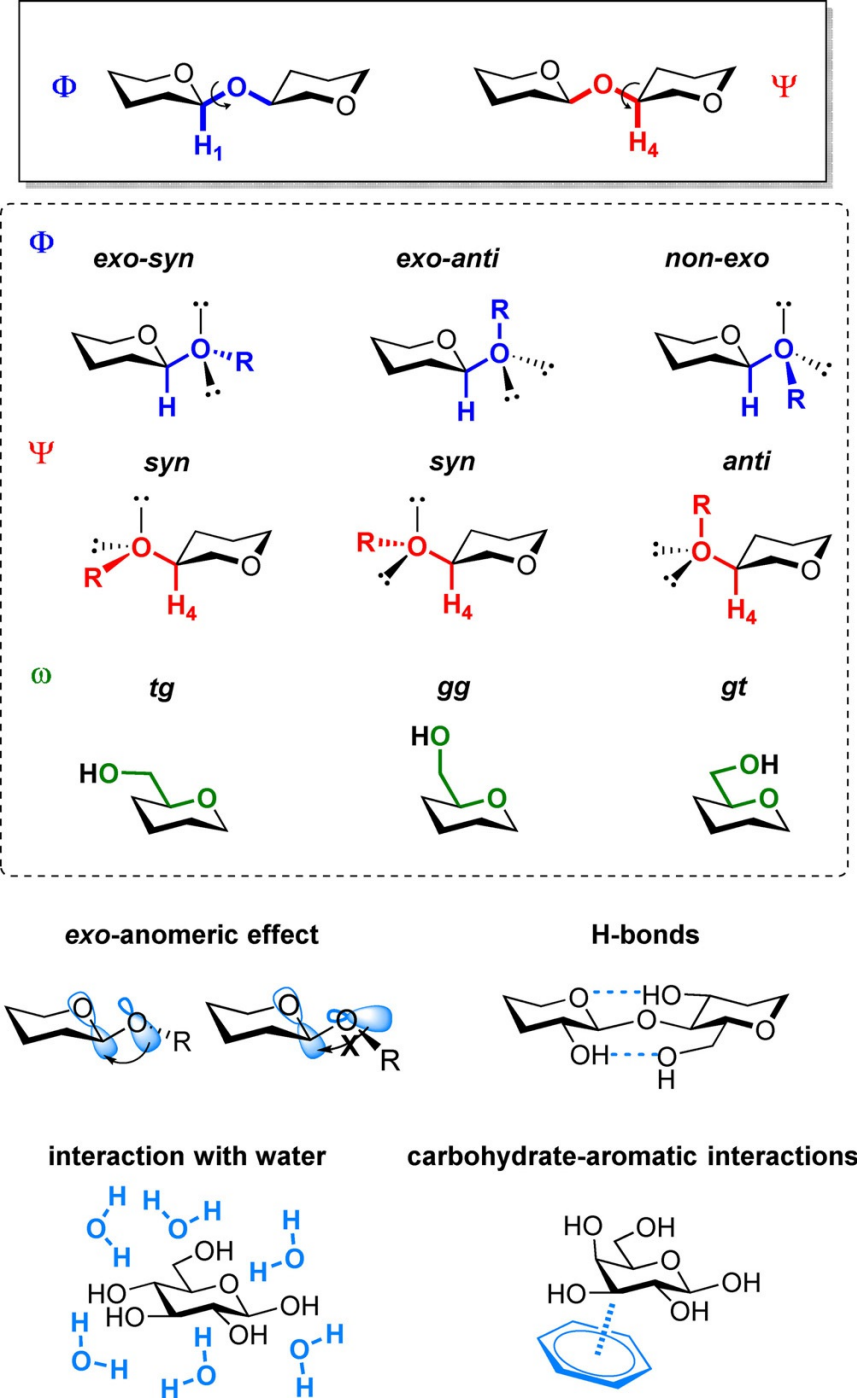
Standard definition of dihedral angles used for the description of a glycosidic bond exemplified for a β‐glycosidic linkage and common interactions that can affect these angles.

When considering conformation of glycans, the monosaccharide unit is generally treated as a rigid block.15a The geometry of the glycosidic bond is of fundamental importance and it is identified by using standard descriptors (Figure 1). Two torsion angles define the relative orientation of the two monosaccharides connected through the glycosidic bond: *Φ* (H_1_‐C_1_‐O_*x*_‐C_*x*_) and *Ψ* (C_1_‐O_*x*_‐C_*x*_‐H_*x*_). For 1,6‐linkages, the additional torsion angle *ω* (O_6_‐C_6_‐C_5_‐O_5_) is required.

Several factors can affect these torsion angles (Figure 1).15a Hyperconjugation between the exocyclic oxygen lone electron pair and the antibonding orbital (σ*) of the endocyclic C−O bond stabilizes the *exo‐syn*(*Φ*) conformation (*exo*‐anomeric effect).^17^ Steric interactions mostly affect the *Ψ* dihedral, favoring the *anti*(*Ψ*) conformer.^18^ Electronic effects can promote particular *ω* geometries (gauche effect).^19^ Hydrogen bonds between hydroxyl groups can stabilize particular conformational states.^20^ Water has a huge effect on the conformational freedom of glycans. Indeed, water can easily disrupt intermolecular hydrogen bonds, resulting in highly flexible conformations, and plays a major role in glycan–protein interactions. Modeling the process of solvation remains a major challenge for computational chemists, limiting our ability to reliably predict glycans’ conformations, and their recognition processes.^21^ Moreover, several glycans bear ionic functionalities that further complicate the description.

Upon interaction with a protein, additional parameters can come into play. Hydrogen bonding and coordination to calcium ions can contribute to carbohydrate binding.^22^ Moreover, despite the high hydrophilicity of glycans, carbohydrate‐aromatic interactions are often involved in carbohydrate recognition.^23^ These noncovalent interactions between two or three CH groups of the pyranose unit and the π electron density of the aromatic ring are able to stabilize particular conformations and favor binding. Stereoelectronic effects also play a role in glycan recognition.^24^

All these factors need to be considered to develop reliable tools to predict glycans’ conformations. Force fields, specifically optimized for carbohydrates, are available and MD simulations have become substantially more accurate.15a, 25 Still, the computational predictions require constant validation with synthetic standards. To date, NMR analysis has offered the best solution with scalar *J*‐couplings and residual dipolar couplings (RDCs) being relatively easy measurements and extremely informative.14a However, glycans’ intrinsic flexibility often leads to an averaged 3D structure, merging the contributions from multiple conformational states.

### N‐Linked‐glycans

**Figure 2:**
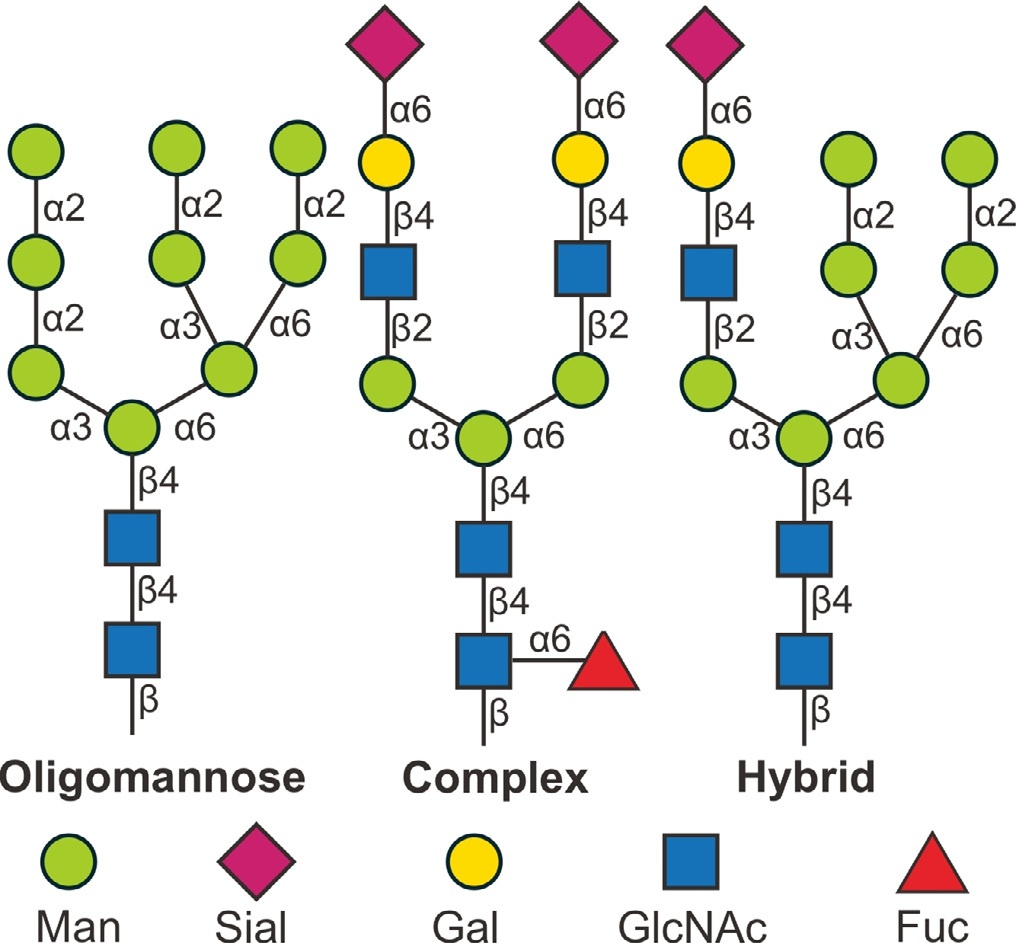
Representative examples of the three main classes of N‐glycans. The monosaccharides are represented following the symbol nomenclature for glycans (SNFG).^27^

N‐Glycans are oligosaccharides covalently linked to secreted and membrane‐bound proteins through an N‐glycosidic bond. They play central roles in the folding, sorting, and transport of proteins as well as mediating cell‐cell interactions.^26^ The N‐glycans chemical structure features a pentasaccharide core motif, consisting of a chitobiose (GlcNAc*β*1→4GlcNAc) and three mannose units. Depending on the residues attached to this core structure, *N*‐glycans are classified into three main categories: oligomannose, complex, and hybrid (Figure 2). Due to their important biological roles, much research has been devoted to their structural analysis.

NMR spectroscopy is the most frequently used method to get insight into the shape and dynamics of N‐glycans in solution. Still, the complexity of these oligomers poses a severe bottleneck for structural studies. In a pioneering work, the three‐dimensional structure of the high mannose‐type N‐glycan domain (‐(N‐acetylglucosamine)_2_‐(mannose)_5–8_) decorating the glycoprotein CD2 was elucidated.^28^ The sample was extracted from Chinese hamster ovary cells. The geometric restraints identified through NOE signals suggested a structural model with one of the glycan arms folded toward the GlcNAc1‐GlcNAc2‐Man3 trisaccharide core. The comparison of ^13^C NMR peak widths shed light on the N‐glycan flexibility, upon interaction with a protein. These results indicated that the N‐glycan does not directly mediate the binding of CD2 to its counterreceptor CD58, since the N‐glycan is oriented in the opposite direction to the binding site of CD2. Instead, the main role of this N‐glycan is to stabilize the folding of CD2, counterbalancing the energetically unfavorable cluster of positive charges arising from five lysine residues.

**Figure 3:**
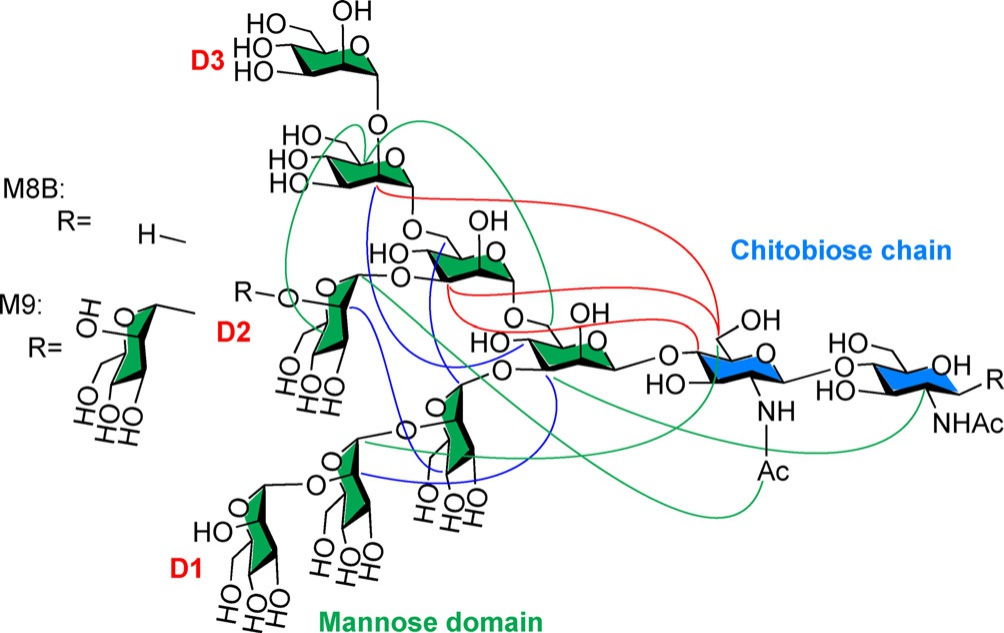
Chemical structure of oligomannose‐type *N*‐glycan M9 and M8B and NOE network. The D2 and D3 branch folds back onto the main chain. NOE signals detected for: both M9 and M8B (red lines), M9 only (blue lines), M8B only (green lines).

Due to the heterogeneity of N‐glycans in biological systems, extracted N‐glycans exists as a mixture of compounds, which leads to ambiguity in NMR peak assignment. This lack of pure and well‐defined glycans hinders detailed structure‐functionality correlations. Genetic engineering provides tools to facilitate glycan structural studies. The biosynthesis of high‐mannose N‐glycans in yeast takes place in the endoplasmic reticulum, where an undecasaccharide Man_9_GlcNAc_2_ (M9) is constructed. Subsequently, an α‐mannosidase yield the decasaccharide Man_8_GlcNAc_2_ (M8B), which is then transported to the Golgi apparatus for further structural modifications. By knocking out the genes encoding for specific enzymes in this route, M9 and M8B were overexpressed and isolated (Figure 3). Moreover, by feeding ^13^C‐labeled glucose to the yeast, isotopic labeling can be achieved, which greatly helps the NMR analysis. Following this approach, it was confirmed that the mannose outer branch (D2 and D3) folds back toward the core chain, similarly to what observed for the above mentioned high mannose‐type glycans in CD2. Notably, compared to M9, a significantly different NOE network and enhanced back‐folding was observed for M8B (Figure 3).^29^

**Figure 4:**
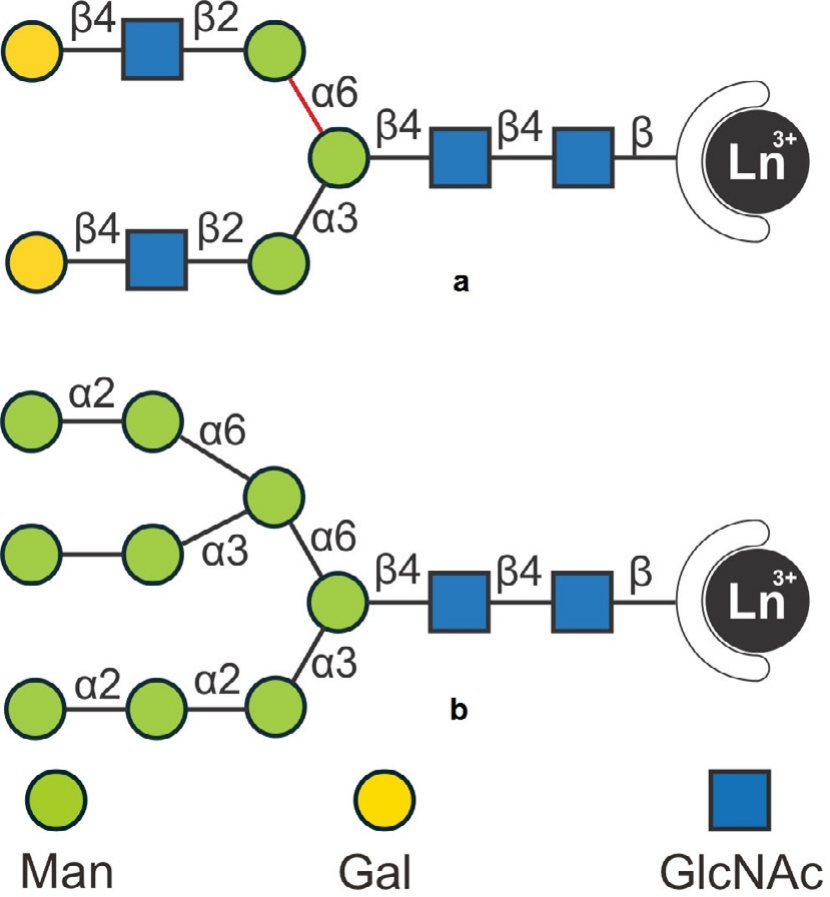
Lanthanide‐tagged N‐glycans for structural analysis. A T‐shaped rotamer at the Manα1‐6Man portion (red bond) was identified as the major conformer. The monosaccharides are represented following the symbol nomenclature for glycans (SNFG).^27^

Further difficulties in the NMR analysis of N‐glycans arise from the multiantennary pseudo‐symmetry that leads to signal overlapping. In addition, NOEs and scalar couplings only afford local structural information (up to 5 Å). For N‐glycans with sizes of several nanometers, analyzing methods able to detect long‐range interactions are required. Paramagnetic lanthanide ions can generate strong chemical shift variations, pseudo‐contact shifts (PCS), and provide global geometric information. A lanthanide‐binding tag was attached at the reducing end of a complex‐type N‐glycan (Figure 4 a). The shift of the NMR signals caused by the paramagnetic ion resulted in 34 ^1^H NMR PCS, that can be interpreted into atom‐atom distances in a range of 30 Å. By computing the obtained conformational information, a T‐shaped rotamer at the Manα1‐6Man portion (Figure 4 a, red bond) was revealed as the major conformer among the five suggested by MD.^30^ This approach was then applied to revisit the structure of oligomannose‐type N‐glycans (Figure 4 b),^31^ supporting the previous observations with a more quantitative description of the glycan dynamics and flexibility in solution.

**Figure 5:**
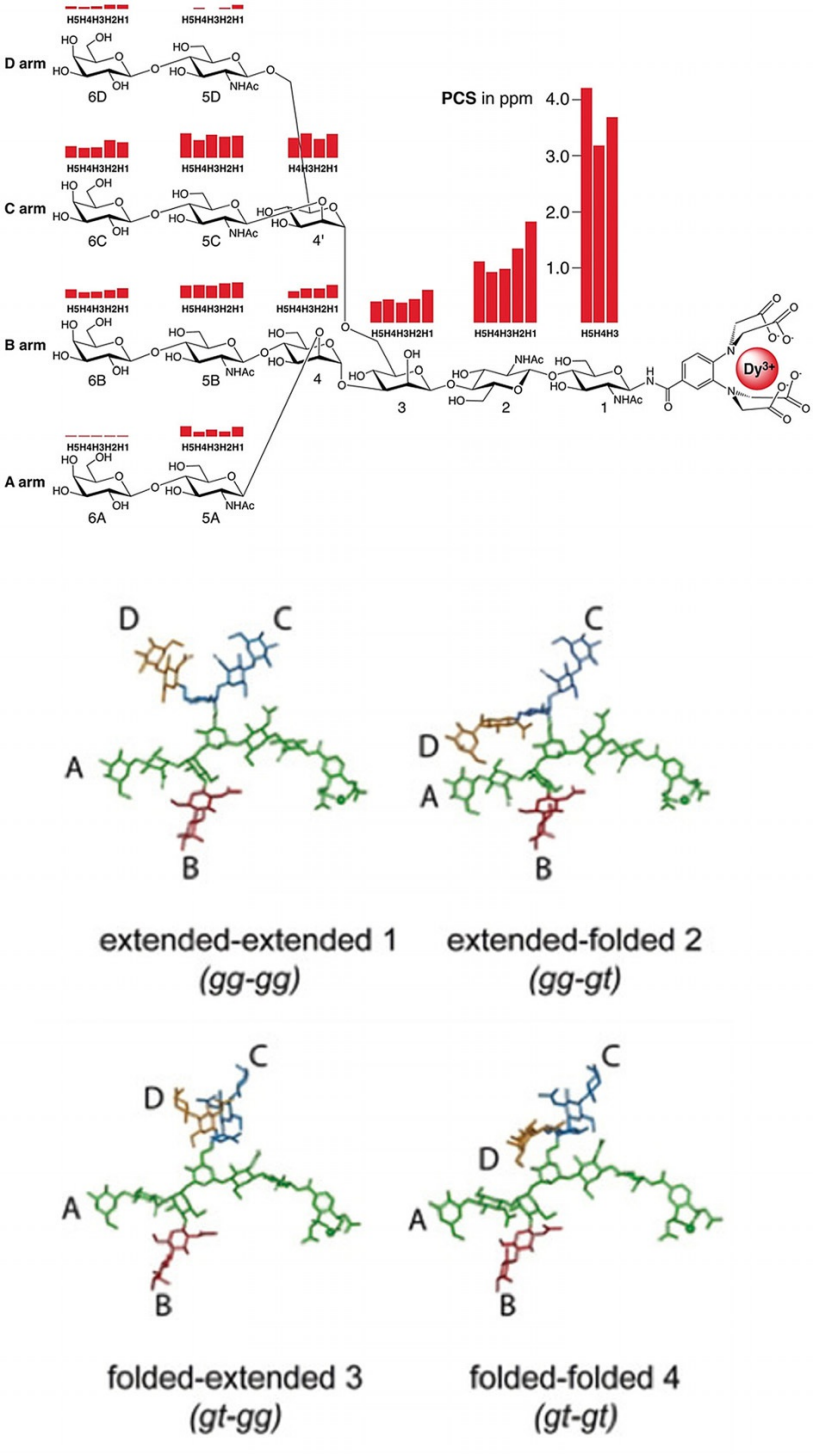
PCSs and minimum‐energy conformations of tetra‐antennary *N*‐glycan as obtained by NMR measurement and MD simulations. Adapted with permission from Ref. 34. Copyright 2017, The Authors. Published by Wiley‐VCH Verlag GmbH & Co. KGaA.

While it is clear that N‐glycans exercise their biological functions through interaction with proteins, the structural basis of this process is not yet solved. Due to the synthetic and analytical difficulties, most studies on glycan–protein interactions are based on fragments of natural glycans. Still, the assumption that the binding behavior of natural N‐glycans can be extrapolated from glycan fragments can be misleading. With the recent advancement in both chemical synthesis and NMR technologies, the binding pattern of natural complex‐type N‐glycans to different lectins can be carefully studied. A collection of synthetic N‐glycans, including several smaller fragments, was synthesized.^32^ Saturation‐transfer difference NMR (STD‐NMR) analysis showed that the interaction between glycan and protein highly depends on the chemical nature of both components and cannot be predicted from simplified mono‐domain models. Lanthanide tags were also employed for the structural study of glycan‐protein recognition.^33^ With this method, the four antennae of the complex‐type N‐glycan can be discriminated and thus information with unprecedented resolution was obtained. The involvement of each individual branch of the N‐glycan in the recognition was described based on PCS (Figure 5). The use of paramagnetic NMR was also applied to the study of the conformation of multiantenna N‐glycans and their interaction with HK/68 hemagglutinin from influenza viruses. This study was enabled by chemoenzymatic synthesis of long‐chain N‐glycans containing poly‐LacNAc and Neu5Ac residues, followed by conjugation with a lanthanide binding tag.^34^

**Figure 6:**
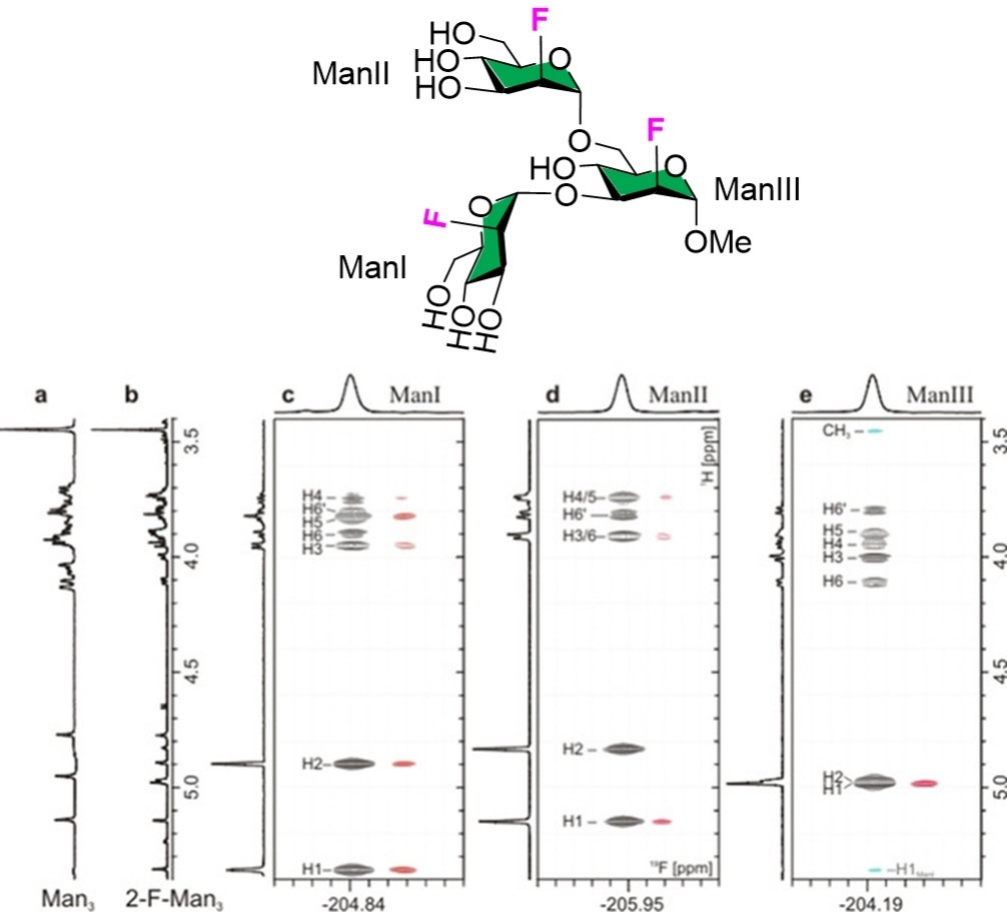
Deoxifluorination breaks the chemical shift degeneracy of glycan NMR and enable epitope mapping. Adapted with permission from Ref. 35b. Copyright 2018, Wiley‐VCH Verlag GmbH & Co. KGaA.

An alternative method to break the chemical shift degeneracy observed for multiantennary N‐glycans relies on the introduction of unnatural and NMR active nuclei. The chemical shift of ^19^F is very sensitive to subtle changes in the chemical environment and spans from approximately −60 to around −220 ppm. Therefore, the substitution of a hydroxyl group with a fluorine atom enables the use of fluorine‐based NMR methods for epitope mapping, as exemplified for the trimannoside core structure (Figure 6). In order to minimize the impact of the deoxifluorination on binding to *Pisum sativum* agglutinin, the modification was installed at the C2 of the mannose rings, following the consideration that the 2‐OH does not participate in this recognition event. 2D NOESY‐TOCSYreF experiments revealed two binding modes in which either mannose I or mannose II are involved. In both modes, the mannose III residue contributes to the binding through collateral effect.^35^

### Histo‐blood group antigens

**Figure 7:**
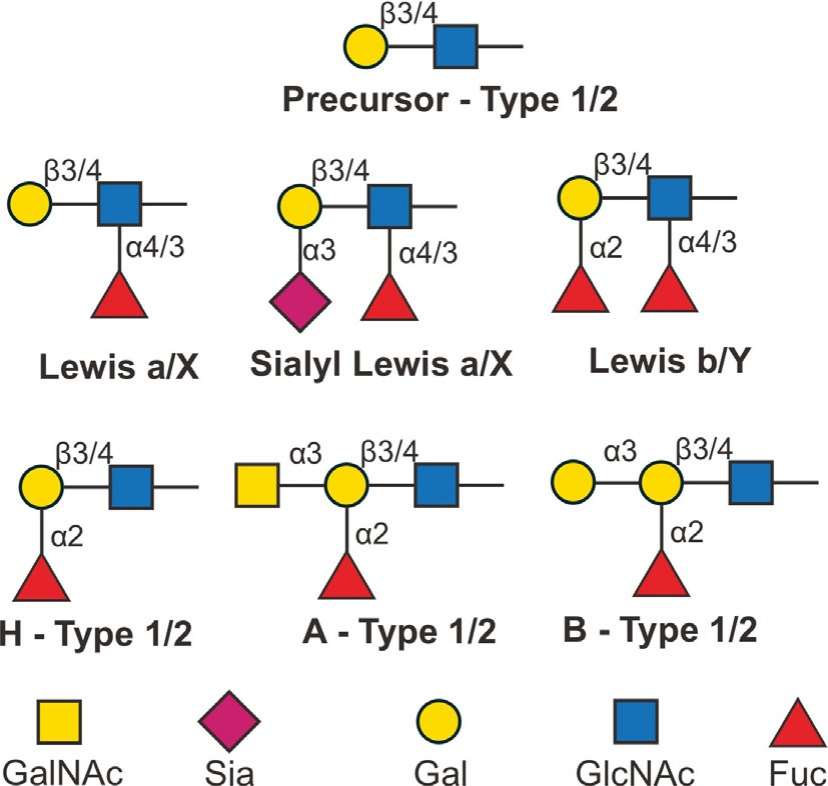
Classification of histo‐blood group antigens. The monosaccharides are represented following the symbol nomenclature for glycans (SNFG).^27^

Histo‐blood group antigens (HBGAs) are a family of oligosaccharides found on the surface of red blood and tissue cells or as soluble antigens.^36^ HBGAs are biosynthesized from two precursors (type 1 and 2 chains) by glycosyltransferases and can be classified as ABH or Lewis antigens (Figure 7). ABH antigens determine the blood phenotype (A, B, AB, or O) of humans and recent studies suggest that they also affect the susceptibility to bacterial and viral infection.^37^ The abnormal expression of HBGAs might contribute to the increased mobility of tumor cells, resulting in poor prognosis.^38^ Structural studies of HBGAs and HBGAs‐protein complex are crucial to unravel the molecular basis of the HBGAs recognition by pathogens.8a, 39

**Figure 8:**
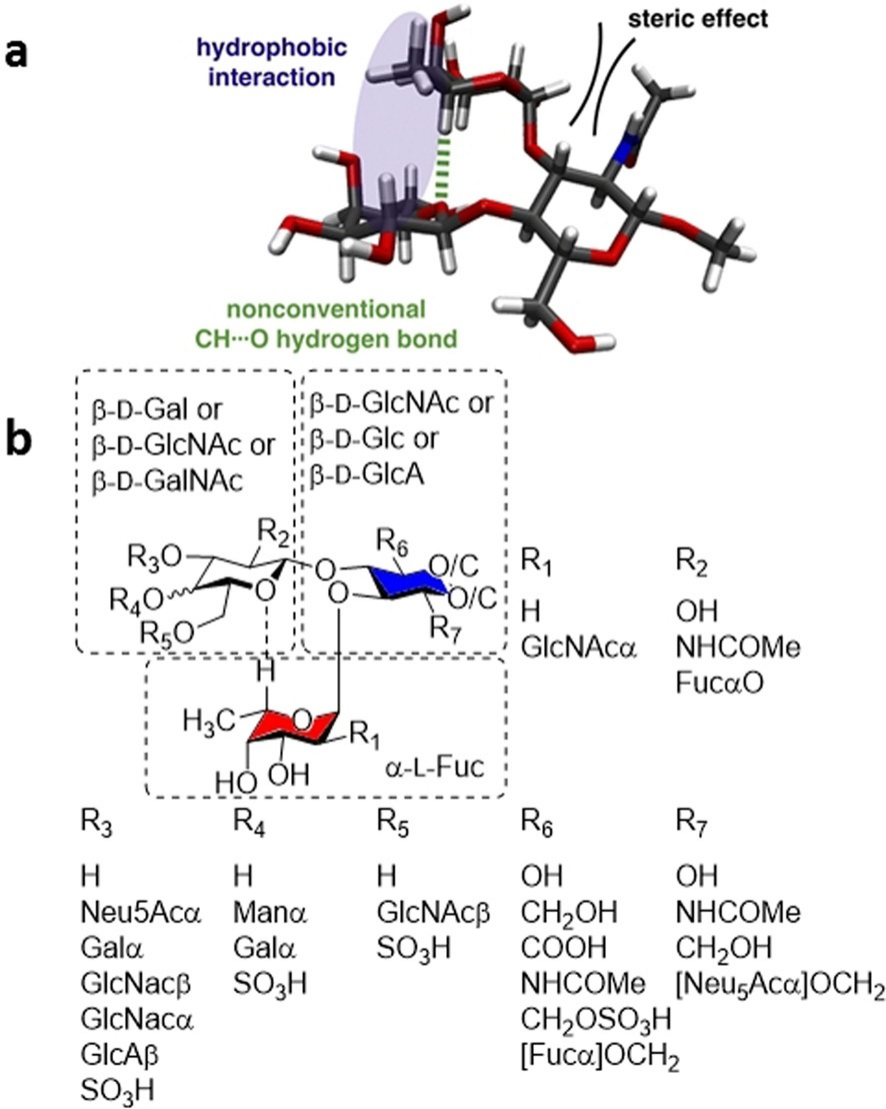
Nonconventional C−H⋅⋅⋅O H‐bonding identified by NMR (a). This is a recurrent secondary structural element in a wide range of fucose containing oligosaccharides (b). Figure 8 a is reprinted with permission from Ref. 42. Copyright 2013, American Chemical Society.

Among the Lewis antigens, sialyl Lewis X (sLe^x^) is the most intensively studied structure due to its vital role in cell‐cell communication, upon interaction with selectins. The core structure, Lewis X (Le^X^), possesses a trisaccharide motif that adopts a defined conformation in solution as revealed by NMR^40^ and molecular dynamics.^40^ Such conformation is stabilized by the *exo*‐anomeric effect, steric compression, and hydrophobic interactions. In 1996, the first crystal structure of Le^X^ was reported, showing the dense network of hydrogen bonds that stabilize its conformation.^41^ To better understand the solution phase conformation, the Le^X^ trisaccharide was synthesized and covalently linked to an isotopically labelled bacterial protein.^42^ This strategy permitted to slow down the oligosaccharide tumbling and, therefore, favored the detection of NOEs. Nine inter‐residue NOEs were detected, while only three could be visualized for the free Le^X^. No NOEs were detected between Le^X^ and the protein, confirming that these results are representative of the conformation of free Le^X^. This study provided important structural information on the torsion angles of the glycosidic bonds and the overall shape of Le^X^. In particular, a nonconventional C−H⋅⋅⋅O H‐bonding between the fucose and the galactose residue was identified and confirmed by the downfield chemical shift of the H5 of the fucose residue (Figure 8 a). Such interaction stabilizes the „closed“ conformation of the Le^X^ trisaccharide motif. The existence of this unconventional C−H⋅⋅⋅O H‐bonding was later confirmed in sLe^x^ with extensive NMR study.^43^ A systematic analysis of several fucose containing oligosaccharides showed that this particular C−H⋅⋅⋅O H‐bonding is a common secondary structural element in a wide range of bacterial and mammalian oligosaccharides and can be generalized with the X‐*β*1,4‐[Fuc*α*1,3]‐Y and X‐*β*1,3‐[Fuc*α*1,4]‐Y description (Figure 8 b).16a

**Figure 9:**
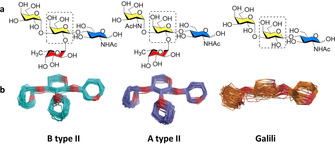
Chemical structures (top) and MD models (bottom) of synthetic glycans showing different flexibility depending on the presence of the fucose residue. Adapted with permission from Ref. 45. Copyright 2019, The Authors. Published by Wiley‐VCH Verlag GmbH & Co. KGaA.

Upon binding to most lectins, the “closed” conformation of Le^X^ is preserved, as confirmed by crystallographic and NMR analysis.^44^ In these cases, fucosylation seems to be responsible for the HBGAs high binding affinity to lectins, by promoting a “pre‐organization” that eliminates the step of conformational selection. To systematically prove this hypothesis, A‐ and B‐ blood‐group tetrasaccharides (type II), as well as their non‐fucosylated analogues were synthesized (Figure 9).^45^ STD‐NMR suggested that the β‐Gal residue (Figure 9, dashed boxes) directly participates in the binding to galectin‐3, while the fucose residue does not interact with the lectin. This proved that Fuc‐containing glycans share the same binding mode with the non‐fucosylated analogues, even though exert higher affinity to the lectin. Thermodynamic and kinetic parameters suggest that the fucose residue contributes indirectly to the binding, reducing the conformational flexibility and so minimizing the entropic penalty of binding.

Similarly, a large entropy contribution drives the binding between sLe^X^ and E‐selectin. Commonly, the interaction between glycans and lectins is entropically unfavorable, but driven by the favorable binding enthalpy.^46^ However, in case of sLe^X^‐E‐selectin interaction, the pre‐organization of sLe^X^ serves as a surrogate for the water cluster present in the binding site.^47^ Upon binding, water is released from the binding site, resulting in an entropic benefit for the overall process. In addition, the pre‐organization of sLe^X^ offers an array of directed H‐bonds increasing the specificity of the binding.

**Figure 10:**
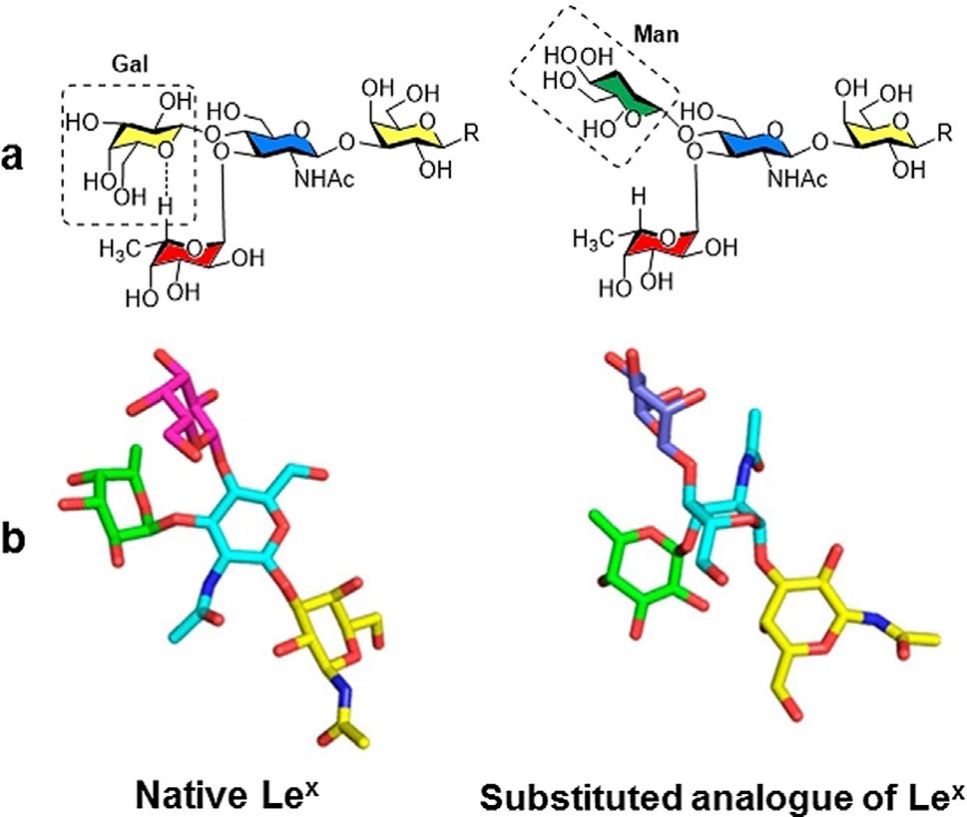
The substitution of the terminal galactose with a mannose unit disrupts the non‐conventional H‐bond that stabilized the “closed” conformation. Chemical structure (a) and 3D model of major conformers (b), reprinted with permission from Ref. 13b. Copyright 2019, American Chemical Society.

In contrast, upon binding with *Ralstonia solanacearum* lectin, distortion of the Le^X^ “closed” conformation was observed.^48^ Several “open” conformers, in which the fucose residue forms H‐bonds with the lectin, were identified by using highly detailed MD simulations of the glycan in water and its interaction with the lectin. This deformation releases the steric hindrance between the galactose residue and a Trp residue of the lectin. Such an adaptive conformation of Le^X^ can be chemically tuned for the design of high affinity glycomimetics. For example, the substitution of the terminal galactose with a mannose unit was shown to disrupt the conformational rigidity of Le^X^ and stabilize the “open“ conformation required for binding (Figure 10).13b The reduced steric hindrance of this unnatural analogue resulted in a 17 times higher affinity for the lectin than the native counterpart.

### Bacterial glycans

**Figure 11:**
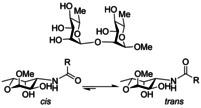
Chemical structure of disaccharide α‐l‐Rhap‐(1–2)‐α‐l‐Rhap‐OMe (top) and 3‐amino‐3,6‐dideoxy‐*α‐* d‐galactopyranose (bottom).

Glycans in bacteria are mostly present as glycoconjugates, such as glycolipids and peptidoglycans.^49^ These glycoconjugates play key roles in the protection of the bacteria from the host immune system and control cellular permeability.^50^ Compared to mammalian glycans, bacterial glycans exhibit a much greater diversity, especially in terms of monosaccharide composition.^51^ These “uncommon” monosaccharides play important roles in the local conformations of bacterial glycans. For example, l‐rhamnose is absent in most mammals, but widely distributes in lipopolysaccharides (LPS) of Gram‐negative bacteria and in capsular polysaccharides of Gram‐positive bacteria. The conformational preference of rhamnose‐containing glycans was studied with a disaccharide model (α‐l‐Rhap‐(1–2)‐α‐l‐Rhap‐OMe, Figure 11, top).^52^ NMR and computational analysis permitted the identification of two preferred conformations in water, existing in a 3:2 ratio. Another sugar motif commonly found in bacterial glycans is the 3‐amino‐3,6‐dideoxy‐*α‐* d‐galactopyranose (Figure 11, bottom).^53^ Particular attention was paid to the conformation of its N‐formyl and N‐acetyl derivatives. The N‐acetyl derivative exhibits a higher preference (Δ*G*°≈−2.5 kcal mol^−1^) for the *trans* conformation compared to its *N*‐formyl counterpart (Δ*G*°≈−0.8 kcal mol^−1^), with a calculated transition energy barrier of around 20 kcal mol^−1^. Quantum mechanics energy calculations suggest intramolecular H‐bonds between the oxygen of the amide and the axial OH4 or the equatorial OH2.

The great variety of monosaccharide composition, substitution, and connectivity observed in bacterial glycans is reflected in great structural diversity that can trigger particular immunological responses. LPS on the surface of Gram‐negative bacteria are based on repetitive polysaccharides that can adopt different shapes.^54^ Due to the structural flexibility, multiple models are generally employed to represent the low energy conformations of these polysaccharides, as in the case of the O‐antigen polysaccharides of *Escherichia coli* O5ac and O5ab.^55^ These structures share the same tetrasaccharide repeating unit connected via different linkages. The conformational preference of these two polysaccharides was studied with NMR methods, including ^1^H,^1^H‐NOESY, and NOE build‐up curves, showing the co‐existence of several different conformers. This structural flexibility could explain the cross‐reactivity of the O5ac and the O5ab antigens in immunological assays.

**Figure 12:**
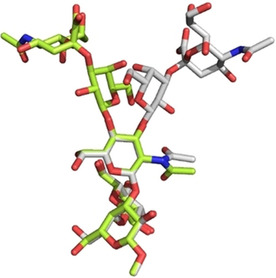
A superimposition of representative 3D structures of the CPS Ia (lime) and Ib (grey) pentasaccharides, with the major conformation around the Neu5Ac*α*2‐3Gal linkage. Reprinted from Ref. 56. Copyright, 2019, The Authors. Published by Wiley‐VCH Verlag GmbH & Co. KGaA.

Similarly, group B *Streptococcus* serotypes Ia and Ib capsular polysaccharides (CPS) share the same monosaccharide constituents with the only difference being one of the glycosydic linkage (GlcNAc*β*1‐3Gal vs. GlcNAc*β*1‐4Gal, respectively). To compare the conformation adopted by these two capsular polysaccharides, the pentasaccharide repeating units were synthesized.^56^ Conformational studies revealed that the difference in GlcNAc‐Gal linkage mainly affects the orientation of the Neu5Ac*α*2‐3Gal branching, with the Neu5Ac*α*2‐3Gal linkages adopting the *exo‐anti(Φ)* conformation for Ia and the *exo*‐*syn(Φ)* for Ib (Figure 12). This different conformational preference might justify the different immunological activity of Ia and Ib capsular polysaccharides.

**Figure 13:**
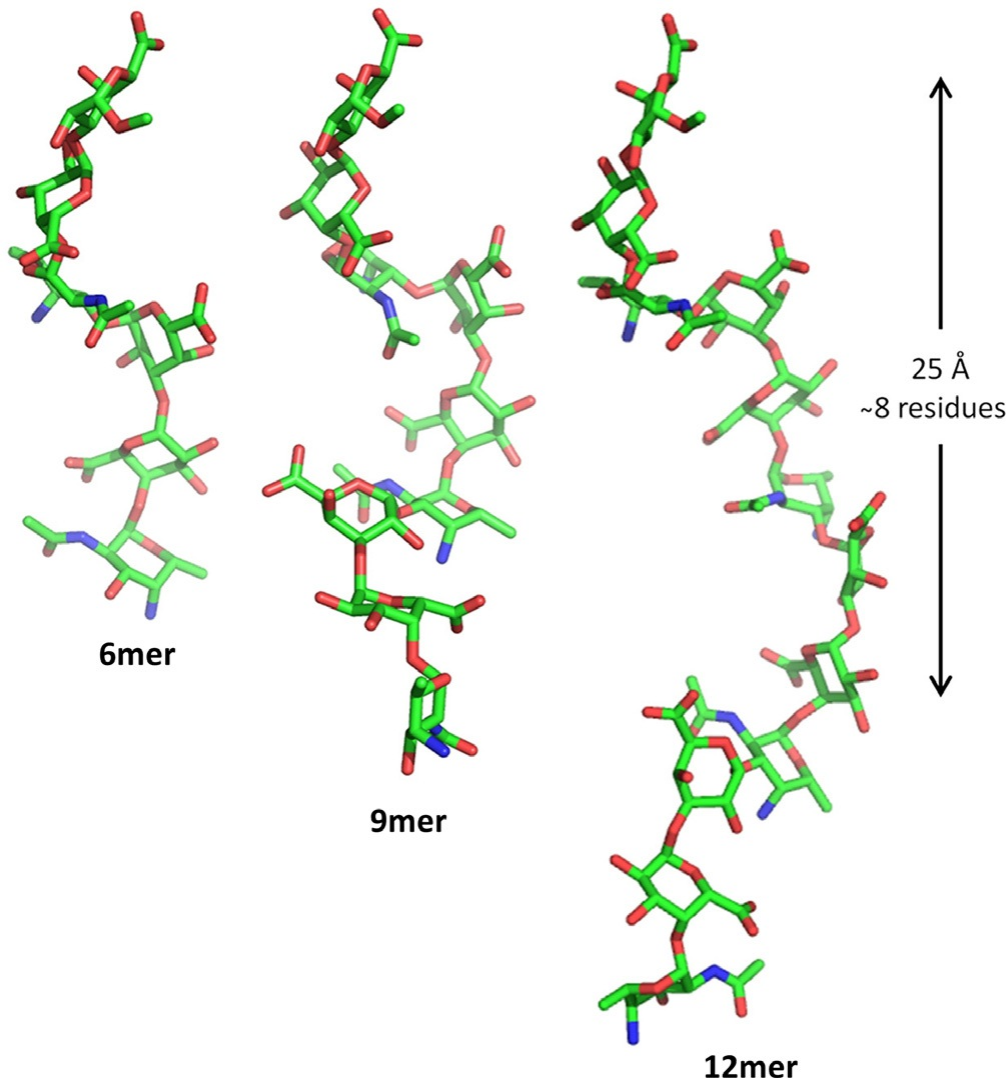
Representative conformations of zwitterionic *Streptococcus pneumoniae* serotype 1 hexasaccharide, nonasaccharide, and dodecasaccharide as obtained by MD simulations. Reprinted with permission from Ref. 13a. Copyright, 2019, American Chemical Society.

Much work has been devoted to correlate the glycan structure to an immunological response.^57^ Zwitterionic polysaccharides (ZPs) from pathogenic bacteria can elicit T‐cell proliferation,^58^ whereas carbohydrates are generally poor T‐cell stimulator.^59^ Conformational studies on extracted ZPs revealed an extended right‐handed helix with positive and negative charges alternately distributed on the molecular surface. This three dimensional structure features a regularly distributed groove, which serves as primary binding domain.^60^ To confirm the correlation between the helical structure and the interaction with antibodies, a collection of zwitterionic *Streptococcus pneumoniae* serotype 1 oligosaccharides was chemically synthetized.13a Structures with lengths of 3 to 12 monosaccharide units were prepared, following a preglycosylation–oxdidation strategy. A strong correlation between the length of sugar chain and the antibody binding affinity was demonstrated, with the highest affinity for the nonasaccharide, able to adopt a full helical turn (Figure 13).

A similar length‐dependent immunological activity was observed for the *Haemophilus influenzae b* antigens.^61^ Chemically synthesized glycoconjugates containing oligoribosyl‐ribitol‐phosphate (PRP) with 4 to 10 repeating units show different immunogenicities, with the tetramer and octamer able to elicit the highest antibody levels. This chain length dependence could be the result of a particular three dimensional structure, best adopted with a certain number of repeating units (i.e., 4 and 8 repeating units).

### Carbohydrate materials

Polysaccharides serve as important biomaterials in nature and are attractive resource of raw material for textile, food, paper, energy, and pharmaceutical industries.^62^ Their primary structure determines their conformational preference and aggregation patterns, which eventually influence the material property. Still, these correlations are far from being established.

Cellulose, the most abundant biomass in Nature, consists of repeating glucose units, connected through β‐1,4‐glycosidic bonds.^63^ Conformational studies based on natural cellulose have yielded considerable knowledge on its 3D structures owing to its high crystallinity, which allows for substantial X‐ray diffraction analysis.^64^ To date, four types of cellulose crystalline forms based on different H‐bonging patterns have been characterized (Cellulose I–IV).^65^

**Figure 14:**
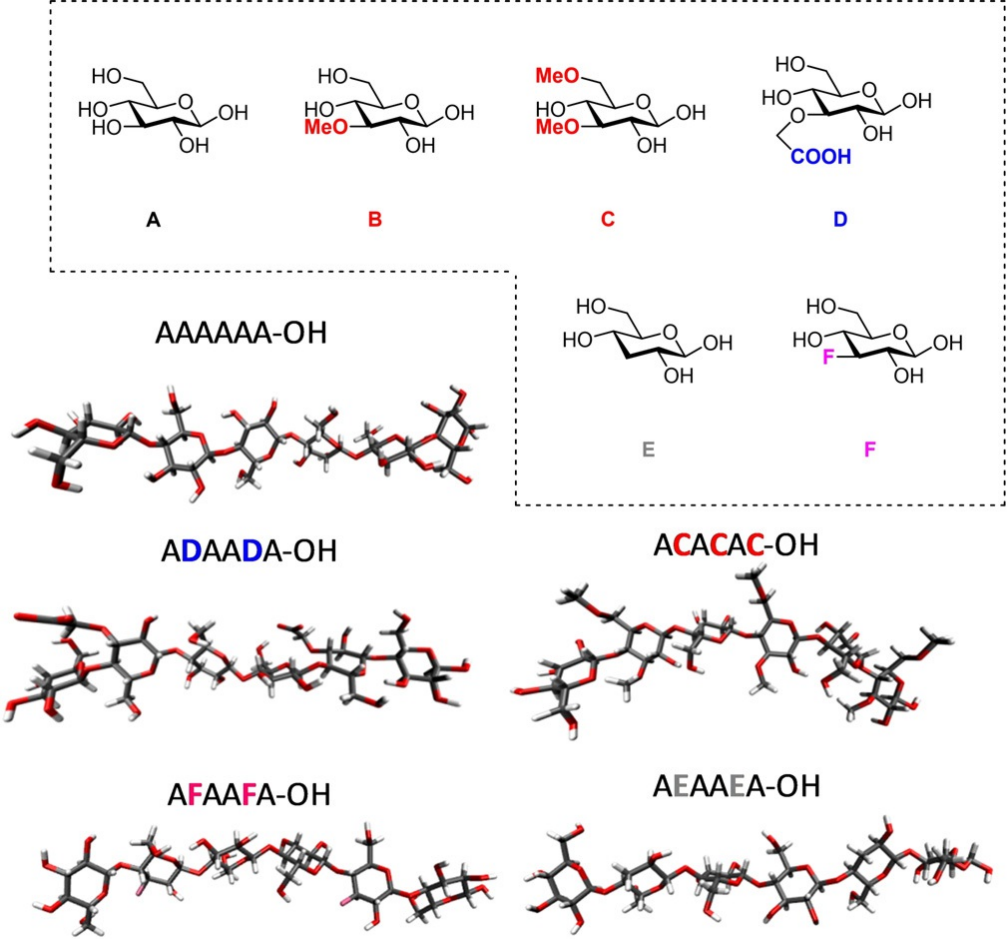
Tailor‐made cellulose oligosaccharides bearing specific modifications and representative oligosaccharides conformations as obtained by MD simulations. Reproduced from Ref. 67. Copyright, 2019, The Authors. Published by Wiley‐VCH Verlag GmbH & Co. KGaA.

Chemical modifications can alter cellulose crystallinity and modulate cellulose properties.^9^, ^66^ However, the lack of regioselective modification strategies yields ill‐defined compounds that prevent detailed structural studies. Automated glycan assembly (AGA) enabled rapid access to cellulose analogues with defined lengths.^67^ A set of “unnatural” monosaccharide building blocks permitted the AGA of chemically modified analogues. With this approach, methylated, deoxygenated, deoxyfluorinated, as well as carboxymethylated cellulose analogues are synthesized with full control over the length (six or twelve units) and modification pattern (Figure 14). The modifications were designed to precisely tune the network of H‐bonds, the steric bulk, and the electronic properties of cellulose.

**Figure 15:**
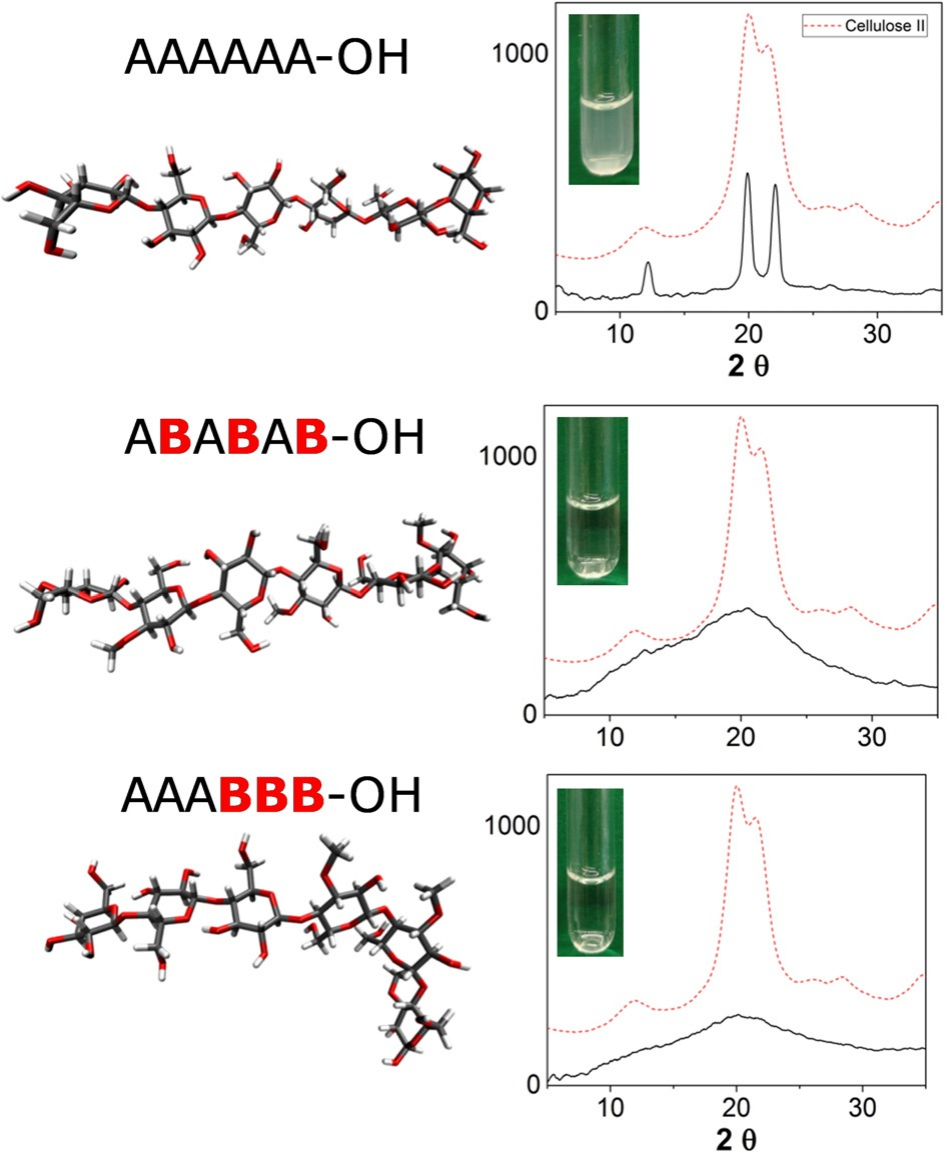
Representative conformations of a cellulose oligomer (A_6_) and two methylated analogues obtained from MD simulations. Powder XRD analysis and solubility test show differences in the aggregations of these compounds. The chemical structure of the monosaccharides A and B is reported in Figure 14. Reproduced from Ref. 67. Copyright, 2019, The Authors. Published by Wiley‐VCH Verlag GmbH & Co. KGaA.

The powder XRD profile of the non‐modified analogues is identical to native cellulose (Cellulose II), indicating that short oligomers (i.e., hexasaccharides) adopt the same arrangement as the polysaccharide counterpart (Figure 15). MD simulations revealed that modifications result in an increased conformational flexibility, which is reflected in an increased water solubility. Notably, methylated analogues with the same degree but different pattern of modification show drastic conformational differences. The analogue with evenly distributed methylation displays a quasi‐linear structure, whereas more compacted geometries are observed with a block‐wise modification pattern (Figure 15). This also affect the solid state arrangement, with a higher “cellulose character” observed for the evenly methylated analogue and a totally amorphous character for the block oligomer. This synthetic approach was then extended to ionic cellulose analogues, bearing amino groups and/or carboxylic acids.^68^ Structural analysis reveals how the charge pattern affects glycan conformation.

**Figure 16:**
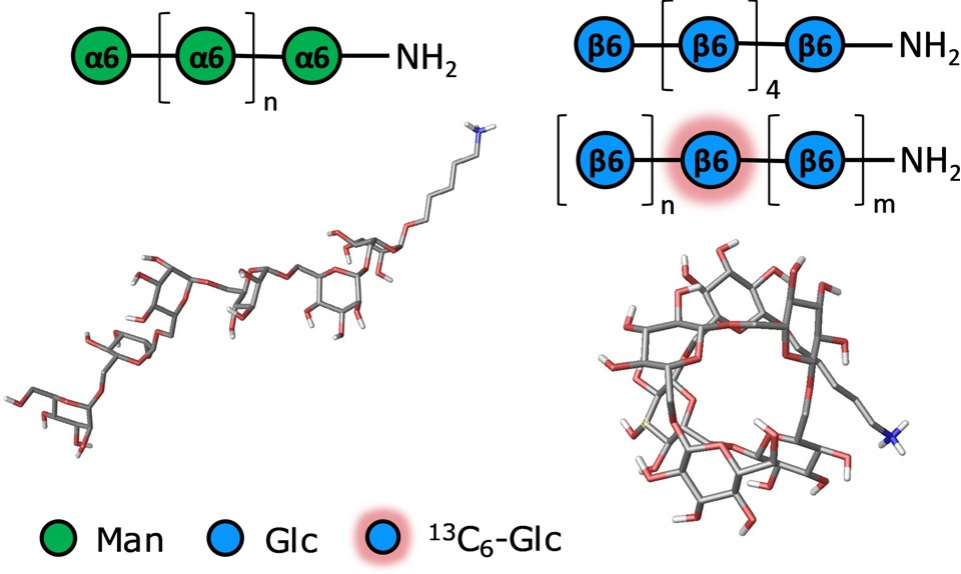
Representative conformations of two oligosaccharides (α‐1,6‐oligomannoside and β‐1,6‐oligoglucoside) obtained by molecular dynamics simulations. NMR analysis enabled by specifically ^13^C_6_‐labeled oligomers confirmed the MD prediction. Reprinted with permission from Ref. 10b. Copyright, 2018, American Chemical Society.

Different classes of oligo‐ and polysaccharides resembling natural and unnatural structures were synthesized with AGA.10b MD simulations and NMR analysis indicate that each oligomer presents a different geometry and flexibility. For example, a α‐1,6‐oligomannoside adopts a flexible linear structure in water, while the analogue β‐1,6‐oligoglucoside displays a more compact helical structure (Figure 16). The synthetic approach permitted the synthesis of ^13^C‐labeled analogues, enabling NMR analysis. A ^13^C_6_‐labeled glucose unit was inserted in specific position of the hexasaccharide chain, allowing for the measurement of *J*‐couplings that confirmed the MD model.

**Figure 17:**
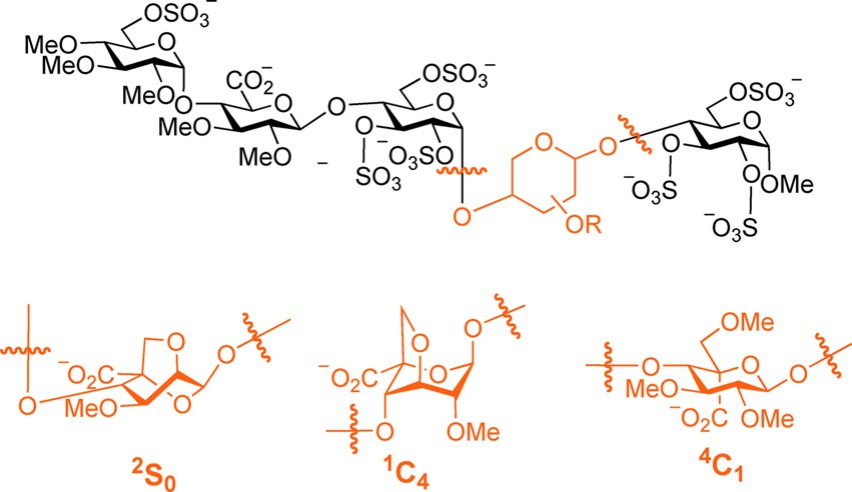
Chemical modifications lock the conformation of l‐iduronic acid, shedding light on the importance of the ^2^ *S*_0_ conformer for biological activity.

Glycosaminoglycans (GAGs) are an important class of structural materials, with vital biological roles in mammals.^69^ GAGs are negatively charged polysaccharides composed of disaccharide repeating units. Hyaluronate (HA), chondroitin sulphate (CS), dermatan sulphate (DS), keratan sulphate (KS), heparan sulphate (HS) and heparin are the most common GAGs. Their structural diversity and conformational flexibility hampers their structural analysis. Chemical strategies to access well‐defined structures are still very labor demanding,^70^ and big collections of related synthetic GAGs are not yet available. In addition, their densely distributed charges drastically influence their interaction with water and metal ions, often resulting in the formation of gels.^71^ Most GAGs are calculated to exhibit left‐handed helices, except for chondroitin and dermatan sulfate, which display a right‐handed helical structure. Fragments of chondroitin and hyaluronan were used as models to identify the highly dynamic intramolecular H‐bonds responsible for their conformation.^72^ Most structural studies on GAGs oligomers are focused on local conformational changes. While most monosaccharides maintain a fixed chair conformation (i.e., ^4^ *C*_1_ and ^1^ *C*_4_), l‐iduronic acid, found in heparin and heparin sulphate, adopts several conformations. NMR analysis of a synthetic pentasaccharide identified an unusual ^2^ *S*_0_ skew‐boat conformer as the major conformer of *l*‐iduronic acid.^73^ To understand the biological meaning of this abnormal conformation, three pentasaccharides were synthesized with the l‐iduronic residue locked into the ^4^ *C*_1_, ^1^ *C*_4_, or ^2^ *S*_0_ conformations (Figure 17).^74^ Only the ^2^ *S*_0_‐locked pentasaccharide strongly binds to antithrombin and potentiates the inhibition of the blood coagulation protease factor. This unambiguously demonstrates that the ^2^ *S*_0_ skew‐boat geometry is necessary for the control of blood coagulation. The importance of the ^2^ *S*_0_ conformer was further investigated by using NMR experiments, crystallography, and computational methods, ultimately resulting in the visualization of the ^2^ *S*_0_ conformer by X‐ray diffraction.^75^

The sulfation pattern also plays an important role in GAGs conformation and dynamics. Fragments of hyaluronic acids with different sulfation patterns (sHA) were synthesized and covalently linked to a surface.^76^ Electrochemical impedance spectroscopy (EIS) suggested that the interaction between GAGs and metal ions is governed by the sulfation pattern rather than by the glycan core.

## Summary and Outlook

For years, the complexity and inherent flexibility of glycans has hampered their conformational analysis. As a result, our knowledge of carbohydrate structures dwarf in comparison to that of peptides and proteins. Recent discoveries have given a new impetus to the structural analysis of glycans, showing that glycans can adopt defined secondary structures.10b, 16a Chemical synthesis has produced well‐defined materials to simplify the analysis.^11^ Several automated techniques are now available for the quick production of collections or related oligomers, as ideal probes for systematic structural studies.10a, 77 Powerful NMR spectrometers have enabled detailed analysis, requiring a minute amount of compounds.14a These data is vital for the developments of reliable computational methods.15a Still, structural analysis of carbohydrates is far from being routine in most synthetic laboratories.

Collaborative efforts between synthetic and analytical experts have proven that even relatively short oligosaccharides can adopt defined conformations. These structural features play an important role in protein recognition and can be exploited for the design of more potent glycomimetics.^12^ To this end, chemical strategies able to disrupt or stabilize particular conformations need to be developed. The conformational space accessible by an oligosaccharide define its aggregation, strongly influencing the carbohydrate material properties.^67^ A better understanding of these interactions could drive the creation of carbohydrate materials by design.

To date, most analytical techniques produce ensemble‐averaged results and might neglect important structural features responsible for a particular activity. Novel single‐molecule imaging techniques can overcome this limitation and allow for definitive structure–function correlations.^78^ A cooperative effort between synthetic, analytical and computational chemists is required to ultimately understand glycans at the molecular level.

## Conflict of interest

The authors declare no conflict of interest.

## Biographical Information

*Yang Yu received his bachelor's degree at Peking University, before joining Prof. Xin‐Shan Ye's group as a master student in 2014. He is currently a PhD candidate at the Max Planck Institute in Potsdam (Germany) in Dr. Martina Delbianco's group. His research focuses on the synthesis and characterization of carbohydrate materials*.

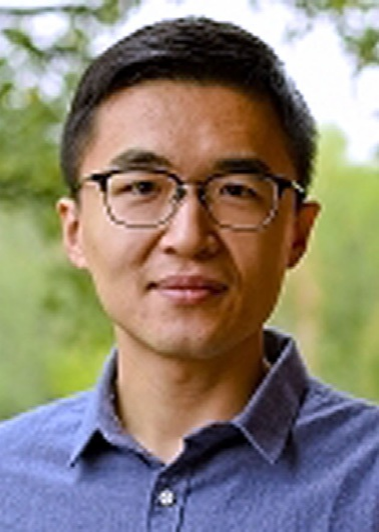

## Biographical Information

*Dr Martina Delbianco obtained her Ph.D. in chemistry at Durham University (U.K.). After two years as postdoctoral fellow in the group of Prof. Peter H. Seeberger, she became group leader of the Carbohydrate Materials group at the Max‐Planck Institute in Potsdam (Germany). Her current research efforts focus on fundamental and applied studies of synthetic oligosaccharide‐based materials*.